# Cellular fingers take hold

**DOI:** 10.7554/eLife.19405

**Published:** 2016-08-09

**Authors:** Yukiko M Yamashita

**Affiliations:** Life Sciences Institute, Department of Cell and Developmental Biology, Howard Hughes Medical Institute, University of Michigan, Ann Arbor, United Statesyukikomy@umich.edu

**Keywords:** spindle orientation, ciliary positioning, *C. intestinalis*

## Abstract

Invaginations in the membranes of embryonic cells appear to orient cell division in sea squirts.

**Related research article** Negishi T, Miyazaki N, Murata K, Yasuo H, Ueno N. 2016. Physical association between a novel plasma-membrane structure and centrosome orients cell division. *eLife* **5**:e16550. doi: 10.7554/eLife.16550

**Image** Membrane invagination projecting towards one centriole in the centrosome (blue dot)

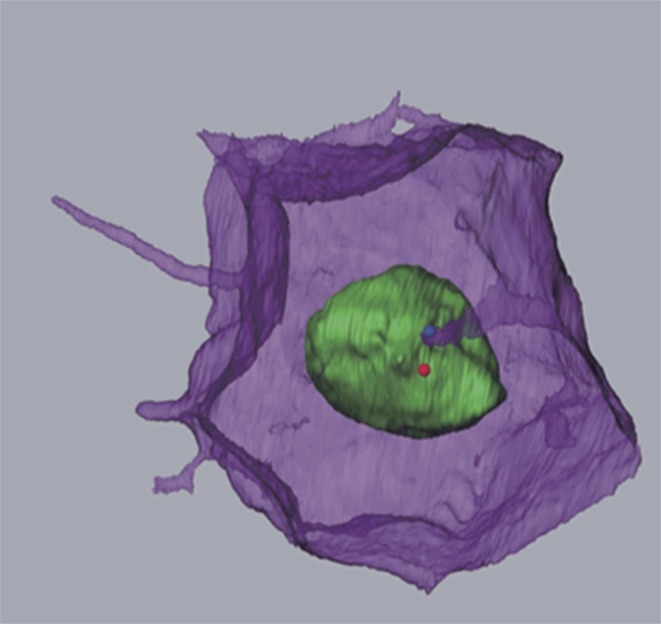

Cells in textbooks tend to have simple shapes, with surfaces that merely separate the contents of the cell from the outside world. However, this is far from the truth. Sperm cells and nerve cells, for example, have quite complex shapes, and the cell surface can play host to a range of organelles and structures, including primary cilia and a variety of other protrusions from the cell membrane (Singla and Reiter, 2006; Kornberg and Roy, 2014; Gerdes and Carvalho, 2008; Buszczak et al., 2016).

Now, in eLife, Takefumi Negishi, Hitoyoshi Yasuo, Naoto Ueno and colleagues report the discovery of a new membrane structure that forms in the embryos of a marine creature commonly called a sea squirt (Negishi et al., 2016). This structure involves a thin finger-like protrusion from one cell’s membrane inserting itself into a pocket (or invagination) formed in the membrane of the cell in front (Figure 1).

Negishi et al. – who are based at National Institutes of Natural Sciences in Japan and Sorbonne University in France – found that the membrane invagination always projects toward an organelle in the cell known as the centrosome. This organelle serves as an organizing center for protein filaments called microtubules. As such, the centrosome has an integral role in the assembly of the mitotic spindle: the macromolecular machine (composed of microtubules) that segregates the chromosomes during cell division.

Negishi et al. suggest that the invagination holds the centrosome in place to ensure that the mitotic spindle is oriented correctly. This conclusion is based upon three lines of evidence. First, the centrosome is closely connected to the tip of the invagination via microtubules. Second, cutting this tip with a laser caused the invagination to quickly retract, demonstrating that it was under mechanical tension. Third, Negishi et al. show that mutant sea squirts that fail to orient their spindles correctly tended to form the membrane invaginations in the wrong direction as well. These mutants included those with defects in a developmental phenomenon known as “planar cell polarity” (often shortened to PCP), which instructs how animal cells in specific tissues become oriented in the same direction. These findings indicate that the direction in which the invagination forms is under the control of the same signaling pathway that controls planar cell polarity.

This circumstantial evidence is strong. However, Negishi et al. were not able to directly test the role of the invagination in orienting the spindle because it regrew rapidly after being cut with the laser. Confirming that the invagination does indeed anchor the centrosome will require further study, in particular to identify the molecular components that govern how the invagination forms.

Further experiments are also needed to answer a number of other questions. For example, how does the membrane know the position of the centrosome in order to project towards it? Although the microtubules that emanate from the centrosome might provide the cue, this mechanism does not explain how the membrane invagination is carved into a thin finger-like tube. Also, is the composition of the invagination different to that of the rest of the cell surface? And if the answer to this question is yes, is there a barrier that keeps the two membranes distinct (as is the case for the primary cilia; Hu et al., 2010)?

Also, how does the invagination orient the spindle? This remains unclear because, by the time the spindle forms and a cell begins to divide, the invagination (like the primary cilium) has been retracted or otherwise removed (Figure 1). Perhaps, the invagination pulls the centrosome when it retracts as the process of cell division begins.

**Figure 1.**
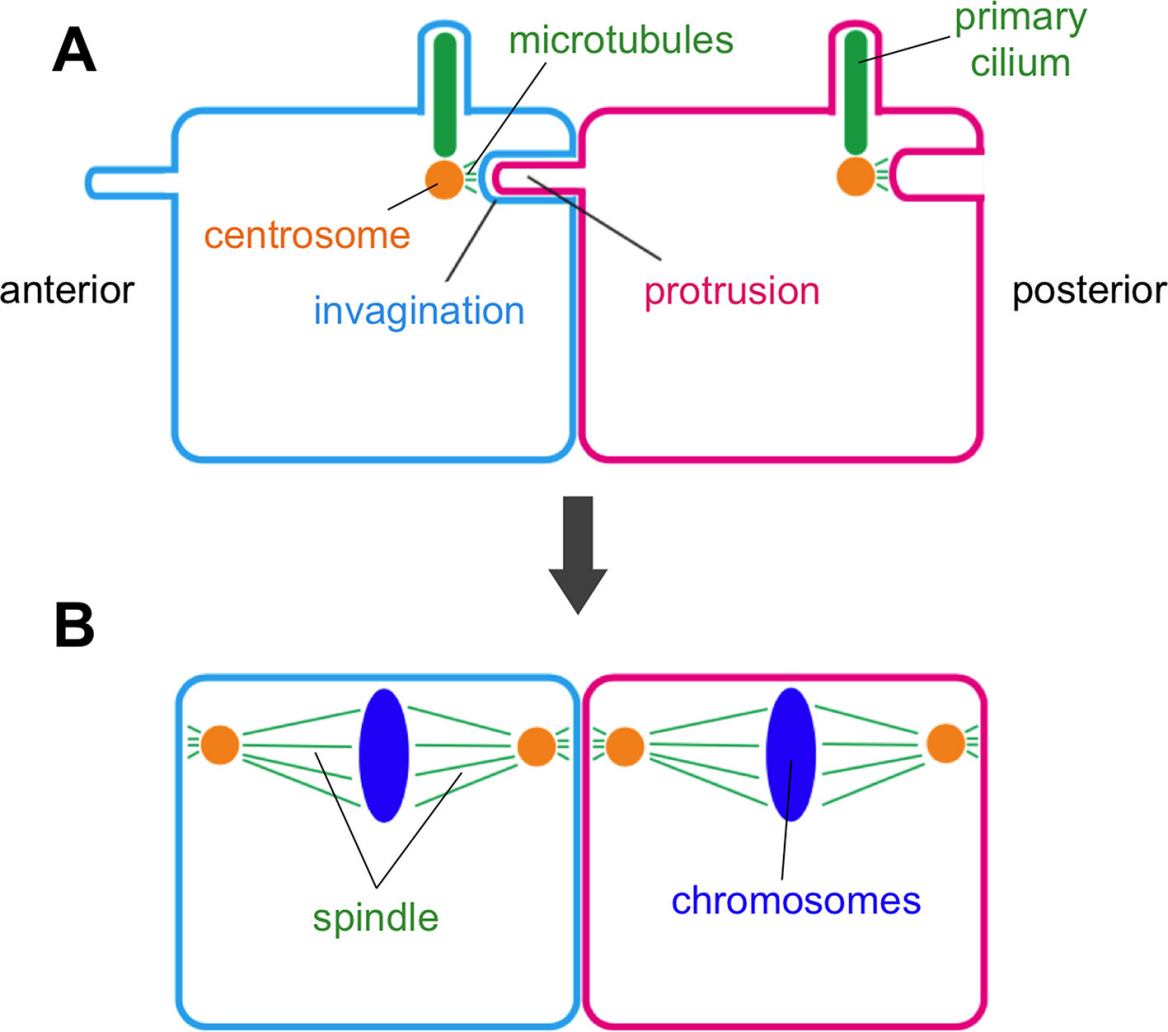
Membrane invaginations and cell division. Schematic diagram showing two neighboring cells in the developing embryo of a sea squirt. (**A**) Before the cells divide an invagination forms in the membrane at the rear (posterior) side of both cells, while a finger-like protrusion forms on the front (anterior) side of each cell and inserts itself into the invagination of the cell in front of it. The tip of the invagination is attached to the centrosome by microtubules. Specifically, the invagination attaches to one of the two centrioles that make up the centrosome – the same centriole that also grows a primary cilium. (**B**) When the cells begin to divide, the invagination/protrusions and primary cilia have disappeared and the mitotic spindles are oriented along the anterior-posterior axis. Negishi et al. propose that the membrane invagination holds the centrosome to orient the spindle.

Many other membrane protrusions have roles in cell signaling: is this the case for membrane invaginations too? Negishi et al. show that the formation of the invagination is clearly downstream of the PCP signaling pathway (Negishi et al., 2016), so it is tempting to speculate that PCP signaling acts through the invagination itself.

Finally, the membrane invagination discovered by Negishi et al. adds to an expanding list of membrane protrusions and organelles. As such we cannot help but wonder how many similar structures have been missed in the cells of other organisms and therefore are still waiting to be discovered.
